# Pneumatic Retinopexy: Confronting Ocular Disease With Visual Art

**DOI:** 10.7759/cureus.29649

**Published:** 2022-09-27

**Authors:** Nealie T Ngo, Andrés Martin

**Affiliations:** 1 Child Psychiatry, University of Toledo College of Medicine and Life Sciences, Toledo, USA; 2 Child Study Center, Yale School of Medicine, New Haven, USA

**Keywords:** bipolar disorder type ii, medical illustration, art, pneumatic retinopexy, retinal detachment

## Abstract

As part of a series of autobiographical case reports about physicians reporting on their own medical afflictions, a psychiatrist copes with a retinal detachment through an artistic collaboration with a medical student. The air bubble injected into the patient’s eye shapeshifts during the six weeks of recovery and becomes the basis for a collaborative artistic project. The series of jointly created images becomes a source of comfort and solace for the patient and of developmental growth for the medical student becoming a physician.

## Introduction

Curtain call

My first patient turned out to be my psychiatry preceptor. To be clear, our introduction did not start out that way. We had met two years before during a research meeting, when I was a second-year medical student and visual artist enthusiastic about bringing together art and medicine. We soon became colleagues across different institutions, with a similar commitment to psychiatry and the medical humanities. It was after a medical emergency on his end and a serendipitous phone call to me later that started us both on a new journey of darkness and beauty toward healing.

For him, the patient,

“Floaters have long been my trusted companions. Eyes open or closed, I have always seen those translucent, shapeshifting, Brownian motion specs in my field of vision. Black dots or hairpins, they are as innocuous as they are perennial. I do not remember a time without them."

He recounted his experience:

“Just as I don't remember a time in which they had multiplied or grown from gnats to flies. I could have blamed exhaustion, were it not for that other unusual (dis) apparition: the lower nasal portion of my right field of vision was intermittently blacked out, curtained off. So intermittent in fact, that I assumed a psychosomatic etiology. I looked in the mirror this way and that, rubbed my eyes, but could find no consistent pattern for either the drone of mosquitoes or the intermittent darkness. I slept, expecting all of it vanished by morning."

"Begone they were not by dawn. In fact, the blind spot had become larger and was fixed in place. As someone with severe myopia (-14.5 0U), I was aware that my eyeball is larger than the norm and elongated in shape, more football than soccer ball. Alas, my poor retina fixed in its ways and size; I knew that at some point its adherence to the globe could give an unfortunate consequence among the nearsighted."

"My team of optometrists were superb psychiatrists. Eye doctors, too. They received the blubbering, worried sick mess that I was with grace and kindness. The prospect of blindness, only partially rational, consumed me. I knew enough from what I had learned in medical school three decades before to be worried, but not enough to address my current spiral into panic. I commiserated and connected, in our native Spanish, with the technician as she applied dilating eye drops. She welled up as I waited for the doctor, seeing the puddle of tears that I was.”

## Case presentation

**Figure 1:**
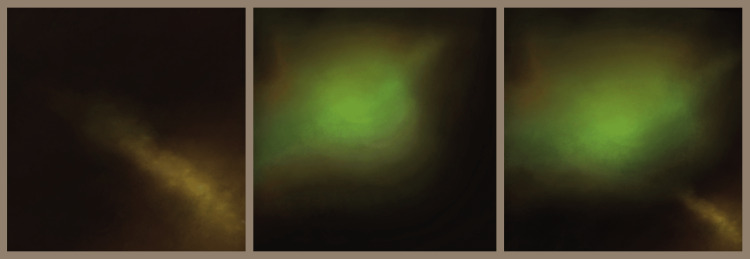
Abstract rendering of a digital retinal image on the day of detachment.

The patient went on to describe his experience, and we started to interlace our different world views (quite literally):

“The optometrist was competent, and thorough, an exemplar of compassion. She made the diagnosis quickly: a retinal tear in the right upper temporal quadrant. While she called a retinal surgeon, I called my wife, and by day’s end I was being examined under lights as bright as I have known. The diagnosis turned out to be correct, to a point. It was not a retinal tear by then, but rather a large retinal detachment. The photograph of my fundus was frightening: a third of my retina had peeled off. A dark fold in an otherwise bright field, it was an ominous and foreboding image (Figure 1). Akin to a tsunami approaching the lights of a city, my optic nerve, macula, and interconnecting highway of thin red vessels sat in the shadow of an impending disaster. Tsunamis are not good. That much I knew."

"I needed treatment on the spot, and the options were not savory. I opted for the first, least invasive, and office-based one. My eye was anesthetized, giving me an unusual, dentist-like feeling. In my eye socket. Not terrible: so far so good. Next, the surgeon inserted a needle-thin wand somewhere in the back of my sclera. The only painful part was when the wand’s tip froze: three short bursts intended to make an abrasion inside the globe, to which the retina could reattach. After experiencing pain in body parts I did not know I own, the surgeon extracted 2.5 mL of anterior chamber fluid before exchanging them for 3 mL of sulfur hexafluoride (SF6) gas.”

Two days after his procedure, I remember waiting for the train, casually scrolling through my email and scanning to see if there was anything important. When I came across one that read,

Hi Nealie, Would u Mind giving me a call today on my cell? Tx

I was immediately sent into a panic. *What did I do? Did I forget something for our research project? Did I miss a deadline? *Fortunately, the phone call had nothing to do with my integrity or responsibilities as a medical student. Unfortunately, it had everything to do with an ophthalmologic emergency that left him with a (healing) detached retina, an eyepatch over his eye, and a foreboding, neon-green wristband that labeled him as a “blindness risk.” Our brief, 10-minute phone call summarized everything that had happened to him and ended with an invitation: could I help illustrate what he was experiencing?

Detached and re-papered

I went on to learn, from his experience (more than from textbooks or PubMed) that retinal detachments can progress rapidly and lead to permanent vision loss if left untreated [1]. In pneumatic retinopexy [2], which is one of the treatments of choice (and the one he opted for), an inert and slow-seeping gas bubble is injected into the posterior chamber of the eye. This bubble presses up gently on the paper-thin retina, helping “plaster” it back to the inner wall of the sclera. Recovery is lengthy but usually uneventful. However, the fluorescent green bracelet slapped on his wrist was the first hint that “uneventful” was unlikely to be in his case:

WARNING. Gas bubble in eye. Use of nitrous oxide (N2O) or changes in atmospheric pressure may cause an increase in intraocular pressure (IOP) resulting in blindness. Consult ophthalmologist on reverse side of bracelet before treatment.

The patient described his experience:

“They began as a collection of smaller bubbles surrounding a large central one. Over the course of six weeks, the collection coalesced into a single bubble. That bubble, hovering as it did in the immediacy of my macula, is the one perfectly sharp thing I have ever seen without correction for my myopia. I had long resigned myself to inhabiting two worlds: one that was 'corrected,' sharp and practical; the other, a blur in which anything unsightly was filtered out, casting me into a personal dreamscape of color and form. Seeing with such sharpness with only one eye, but not both, was a profoundly disorienting experience."

"Wearing a single eyepatch was disconcerting at first, as the contrasts in sight between my left and right eyes were too jarring. Patching both eyes was unexpectedly soothing. Under the cover of darkness, there was little I could see besides the alternating shades of gray and pitch black in the background, and the bubbles’ changing contours and contrasting edges of white, silver, and black."

"As I started loosening the edges sealing the eyepatch in place, light began seeping in. I then began removing the patch entirely, intermittently at first. Through closed eyelids, I looked at progressively brighter sources of light. The sun was best of all, igniting a black and white landscape with warm hues of yellow, orange, and red. Despite my poor draftsmanship, I kept a sketching log of my findings: If nothing else, I would document this unprecedented time. I would strive to remember. I was enthralled under these daily metamorphoses. First the contours, then the colors, finally the crash. Eventually, the bubble dissipated into thin air, quite literally.”

As the new illustrator-on-call, I was thoroughly stumped. *Bubbles? Halos? Metamorphosizing? *My art portfolio consisted of drawing characters and creating comics, stringing together visual narratives and text to tell concrete stories. This assignment was as far from my usual as I could get. It was heading, instead, into the world of abstract art, a realm I was only superficially familiar with.

**Figure 2:**
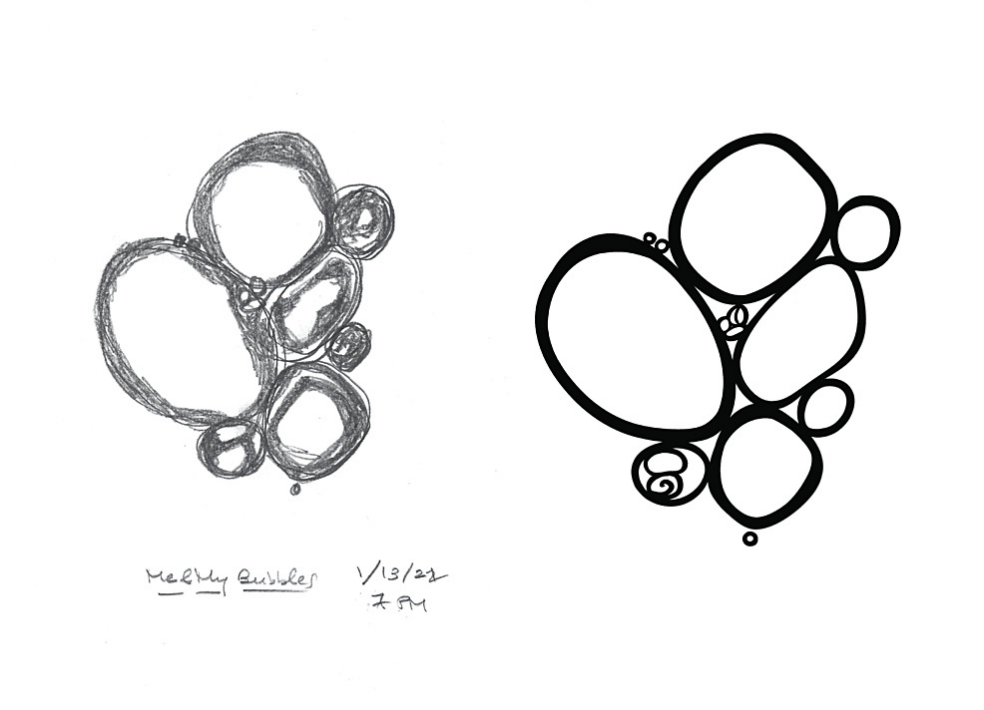
Depictions of intraocular bubbles on the day of detachment: by the patient (left) and by the author (right).

As unconfident as I was at hearing the initial topic, art was still my métier, a soothing, constant companion in a life that has been filled with science, objectivity, and rigidity. I had no idea how, but I wanted to do this. When I received his first collection of sketches, six pencil drawings meticulously labeled with the dates and times seen, along with scattered comments throughout, I realized that my collaboration was not purely mechanical. Over the course of six weeks, each of the 15 sketches I received in my inbox became an additional invitation to become a witness, and eventually, a companion in what would be a complicated journey toward healing. Each sketch I saw became one that we both shared, and one fewer sight that he experienced alone. I began the illustration process by outlining all the bubbles in crisp, black ink, trying to capture an overwhelming sharpness seen for the first time (Figure 2).

**Figure 3:**
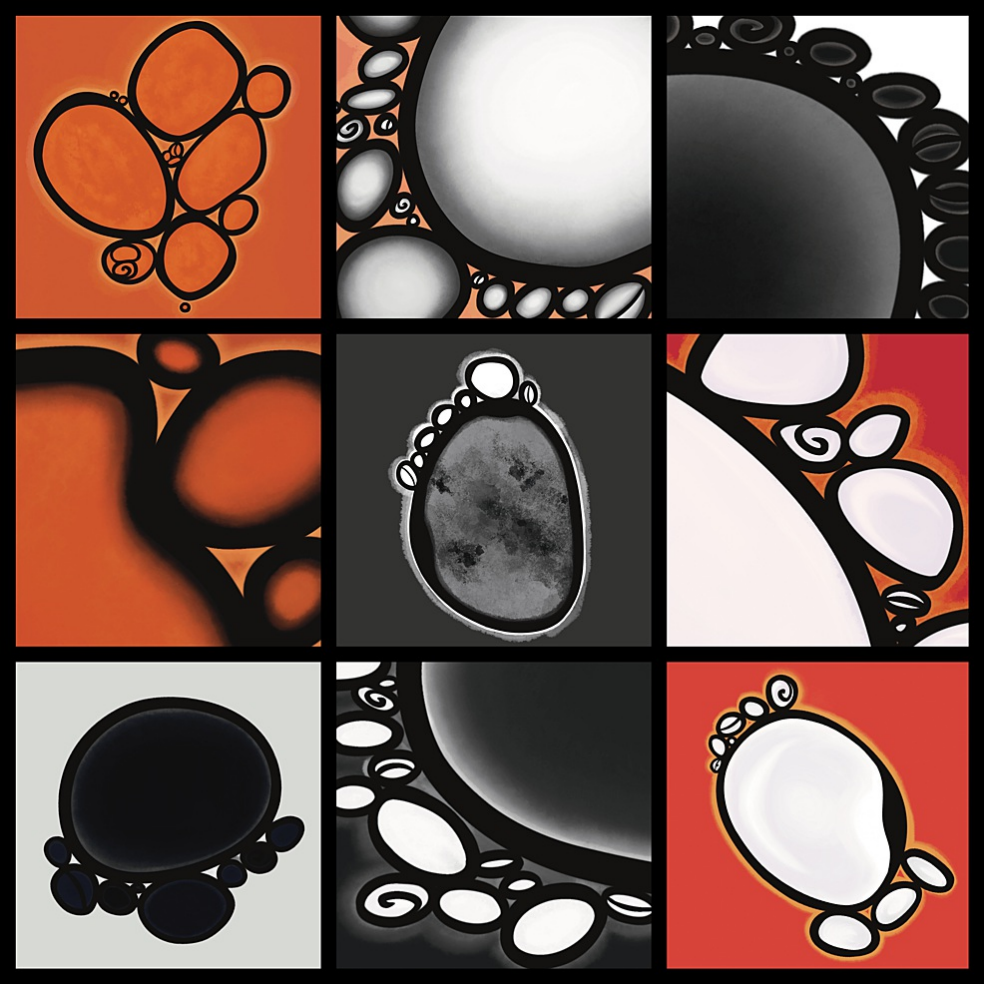
Progression in the shapes, contours, and colors of bubbles over the first three weeks of recovery.

Then came color; we moved from muted gray and black to vivid and luminous bursts of marigold and ember. Each palette brought with it a cascade of unique emotions: alarm, serenity, and awe. As the bubbles coalesced and developed different shades and hues, we found ourselves turning to art, borrowing vocabulary from the world of artists, to describe what he saw in his mind’s eye. Living in New York City at the time, I ventured to the Whitney and the Guggenheim Museums, trying to find inspiration. After months, I finished our first set of paintings: nine panels of bubbles (Figure 3).

However, we both worried: 

“It was not just my retina that was detached; so was I in my retreat."

"I may have been a good patient: respectful, adherent, and grateful. But I was terrible as a convalescent: needy, fragile, dependent, and irritable: gifted at catastrophic thinking. The visual shutdown compounded a lifelong penchant for ruminative introspection. Exercise ground to a halt: running and cycling were too bouncy for my eye; swimming beset by the threat of centrifugal force. The bubble’s fragility required it to be swaddled in stillness. Even walking became unnaturally meticulous. Stray branches from poorly groomed trees could spell disaster by lancing a healthy eye burdened by trying to navigate three-dimensional space on its own. I recoiled from social contact. I was aware of the bore, and the burden, I had become to those closest to me. But perhaps not all was lost: my brain and my mind were healthy."

"I don’t make the statement lightly. The fact that my psychiatric condition was stable was of central importance to my healing process. I had struggled for much of the preceding year with the relapse of a lifelong mood disorder which had undergone its own recent metamorphosis. But for the three months preceding the detachment, and for the duration of its treatment and recovery, things had quieted down, medications stabilized, and normal function returned. My retina may have been torn and my body deconditioned, but my mental health was once again intact.”

During this process, he complimented the sketches with emails related to the bubbles: random excerpts from poems, YouTube links to songs and audiobooks, and forwarded emails from others involved in his healing process. It became normal to see one or two new emails pop up on my computer in-between classes during the day. Then came the texts and voice memos, which at times flooded my phone at unprecedented rates, at unexpected times. Leaving for a 30-minute dinner only to come back to 25 combined text messages and voice memos became something I was unsure how to navigate. There were even days when I would wake up to anywhere from 10 to 15 texts, sent somewhere between 1 and 2 A.M.

This project was already challenging artistically, but this new layer of ambiguity only added to the weight. How to interpret these messages? Were they sent from a source of inspiration or of stress? From the beginning, he had been open about his mental health, both past and present [3,4]. Our formal check-ins via Zoom (San Jose, CA) somewhat quieted my concerns and led me to the conclusions that (1) he was an extremely productive person, supposedly on prolonged bed rest for a detached retina, and (2) art was as much escape as solace for both of us. And so, I continued.

## Discussion

All clear

Time gave us perspective and relief, as well as new concerns:

“Six months after my detachment, I found myself visually stable but psychiatrically ill once again. The first signs of my impending descent were not mood symptoms. Rather, they manifested through my constant fear of a new detachment. Medically cleared by my ophthalmologist, I was traveling abroad for the first time since the pandemic began and since the detachment. I was dockside with dear friends, ready to take a ferry to a secluded island, an optimal destination for winding down. As I approached the gangway, I saw what I perceived as new and particularly large floaters, more flies than mosquitoes. I panicked, I cried, I fell apart; I could not take that ferry and be stranded in an island without tertiary care."

"My surgeon back home was reassuring over the phone but could not be certain without examining me. That wasn't good enough; I knew I would not be able to tolerate the uncertainty. And so began the quest for a retina specialist abroad. My fortune could not have been greater: examined a few hours later by an experienced specialist with superlative clinical skills. Under the lamps, his silence was a gift: 'Unless I say something while examining you, please assume that everything is OK.' Silently leaning in, a consummate therapist by honorary degree. It all checked out: no new detachment."

"I am uncertain if this psychiatric relapse was a new recurrence or the continuation of earlier instability that I did not recognize at the time. The difference is academic, trivial. What is not, however, is the fact that I went on medical leave: an outcome unimaginable to this confessed workaholic and hospitalist. I realized that the enforced darkness, the frailty, and the withdrawn convalescence had all contributed. Apart from the constant fear of blindness in one eye. The detachment may have been a warning: ‘slow down, you're coming apart at the seams.’”

**Figure 4:**
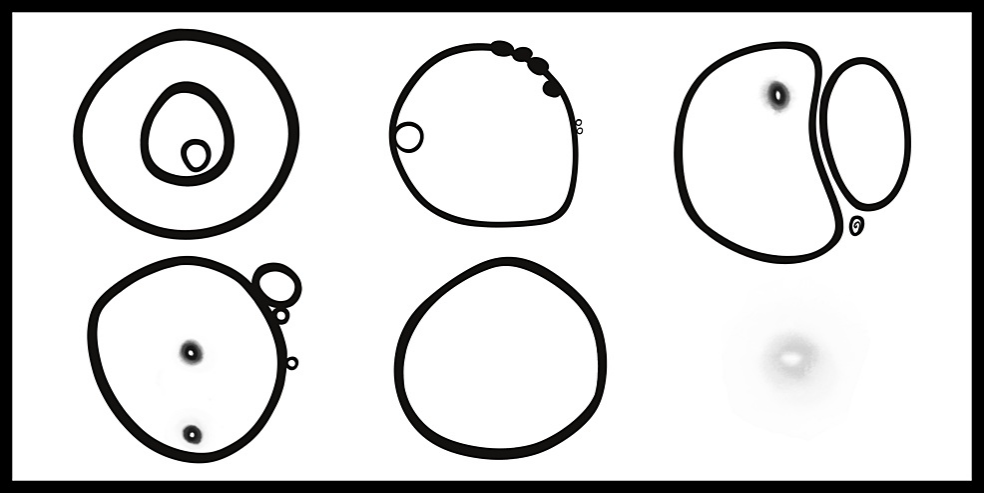
Progressive coalescence toward a single bubble, which once gone, left larger floaters behind.

After his trip abroad, I received fewer messages related to our project. My questions were answered after hearing what had happened, what concerns he and his psychiatrist had for his recent state of mental wellbeing, and that his subsequent plans were to focus solely on his own mental health for a while. The frenetic volume of communication gradually decreased to a new level of what I now understand to be his baseline. In addition, we began having candid conversations about mental health and illness, a topic that many days, included his own. In the months that followed, our project took on a new meaning. It was still a documentation of an “unprecedented time,” but now with multiple interpretations of the phrase. I continued my illustrations, this time creating a second, different set of paintings to reflect this new shift in his journey (Figure 4).

He continued to have challenges:

“Frightened by flashback floaters, which were no longer my innocent companions but potential harbingers of disaster, I wanted them gone. I learned of two surgical approaches, both involving extraction of the vitreous, that could give me crystal clear vision. I wanted the surgery but learned that neither one was entirely risk free: removal of the vitreous can detach edges of the retina, leading to a risk for serious complications, including a full detachment. I passed on the vitrectomy."

"No crystalline view for me then. No life without floaters. Nothing is perfect, certainly not my vision, which has never been nor aspires to being. I have learned to embrace the imperfection. I celebrate it. I can see. I can see beauty. I can see ugliness. I can see it all. My vision restored, warts and all. Oh the joy of being able to see the warts."

**Figure 5:**
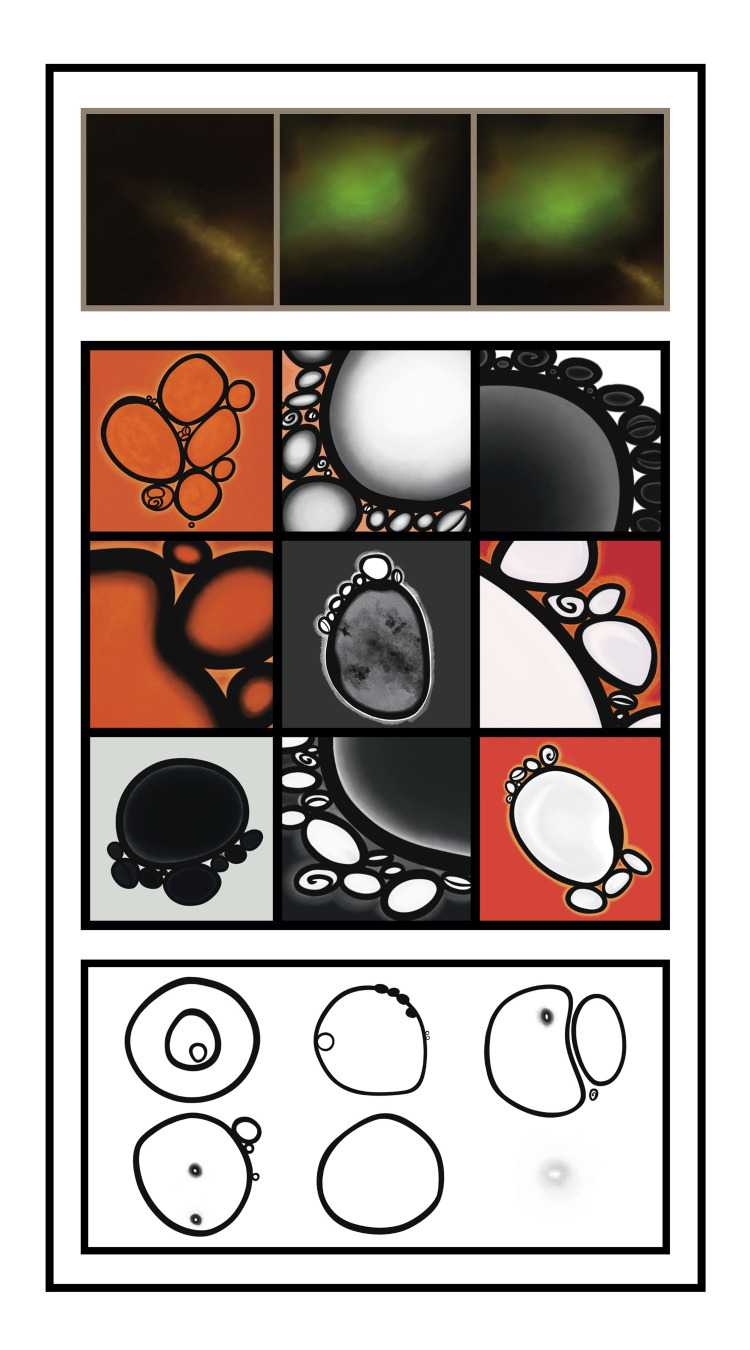
From the inside looking out: abstract rendering of a retinal detachment over the course of six weeks, as viewed from the perspective of the patient.

"At a time when my sight failed, your gift let me see. We created art together, and hope. [5] Thank you, Nealie, for your vision” (Figure 5).

## Conclusions

Art can be a tool of healing, and in his case, I believe it was. He has told me that his recovery would have been different without our many exchanges and creations. Crafting the images he was seeing, contours first, colors later, gave him a sense of control during a time when he had none. He was no longer a passive vessel of the bubbles and their transformations. He came to understand their beauty, and I helped translate these symptoms into documents he could externalize and track his recovery with. These images and the process of making them together gave him reassurance and hope.

Being let into another’s life is among the most rewarding aspects of medicine and among the most instructive. I believe that this process has helped me understand that as a clinician, my ears are just as important as my eyes. I tried to listen and not be judgmental. I did not provide unwarranted reassurances nor unsolicited therapy. I simply reassured him that I welcomed his reflections. This experience has meant a lot to me. I have not yet processed it fully, but know it is helping steer me somewhere important in my development as a future psychiatrist, and for that joint vision, I will forever be grateful.
